# An Overview of Indian Biomedical Research on the Chikungunya Virus with Particular Reference to Its Vaccine, an Unmet Medical Need

**DOI:** 10.3390/vaccines11061102

**Published:** 2023-06-15

**Authors:** Muhammed Muhsin Varikkodan, Faisal Kunnathodi, Sarfuddin Azmi, Tzong-Yuan Wu

**Affiliations:** 1 Department of Bioscience Technology, College of Science, Chung Yuan Christian University, Chung-Li, Taoyuan City 320314, Taiwan; muhammedmuhsinv@gmail.com; 2 Scientific Research Center, Prince Sultan Military Medical City, Riyadh 11159, Saudi Arabia; sazmi@psmmc.med.sa; 3 R&D Center of Membrane Technology, Chung Yuan Christian University, Chung-Li, Taoyuan City 320314, Taiwan

**Keywords:** chikungunya virus, CHIKV, outbreaks, chikungunya vaccine, chikungunya research, biomedical research

## Abstract

Chikungunya virus (CHIKV) is an infectious agent spread by mosquitos, that has engendered endemic or epidemic outbreaks of Chikungunya fever (CHIKF) in Africa, South-East Asia, America, and a few European countries. Like most tropical infections, CHIKV is frequently misdiagnosed, underreported, and underestimated; it primarily affects areas with limited resources, like developing nations. Due to its high transmission rate and lack of a preventive vaccine or effective treatments, this virus poses a serious threat to humanity. After a 32-year hiatus, CHIKV reemerged as the most significant epidemic ever reported, in India in 2006. Since then, CHIKV-related research was begun in India, and up to now, more than 800 peer-reviewed research papers have been published by Indian researchers and medical practitioners. This review gives an overview of the outbreak history and CHIKV-related research in India, to favor novel high-quality research works intending to promote effective treatment and preventive strategies, including vaccine development, against CHIKV infection.

## 1. Introduction

Chikungunya fever (CHIKF) is a virus-borne illness transmitted by mosquitoes that is brought on by an alphavirus from the Togaviridae family. This virus is classified as an enveloped virus with positive-stranded RNA, and the primary vectors are *Aedes aegypti* and *Aedes albopictus* mosquitos. The term ‘chikungunya’ is derived from the root word ‘kungunyala’ of the Kimakonde language, and alludes to the bending posture of patients infected with the virus [1]. Like most tropical infections, the Chikungunya virus (CHIKV) is frequently misdiagnosed, underreported, and underestimated; it primarily affects areas with limited resources, like developing nations [2]. It has become a hazard to public health over the past 20 years, contributing to certain fatalities and substantial disease-associated morbidity [3]. Infection with CHIKV results in a feverish disease like the Dengue virus (DENV) and causes symptoms such as high body temperature, muscle and joint discomfort, flaccid limb weakness, headache, nausea, exhaustion, vomiting, conjunctivitis, rash, and is very rarely deadly in humans [4,5,6]. The key genotypes of CHIKV have been classified as Asian, East-Central-South-African (ECSA), and West African, based on their geographical distributions and all three variants can produce CHIKF in humans [7].

The very first report on CHIKV was from Tanzania, an eastern African country, in 1952 [8]. Later, it was reported from most parts of the African continent including Cameroon, Benin, Senegal, Angola, Burundi, the Democratic Republic of the Congo, Kenya, Madagascar, Gabon, Nigeria, Guinea, Sierra Leone, Liberia, Malawi, Uganda, southern Africa, and Sudan [3,9,10,11,12,13,14,15,16,17]. Subsequently, CHIKV outbreaks have been reported in many Asian countries including Pakistan, Bangladesh, Myanmar, Philippines, India, Sri Lanka, Bhutan, Saudi Arabia, Cambodia, Singapore, Maldives, China, Thailand, Indonesia, Taiwan, Laos, Vietnam, Malaysia, Timor and Yemen [18,19,20,21,22,23]. Since 2004, CHIKV outbreaks have increased in frequency and extent. Primarily as a result of a viral adaptation caused by a point mutation in the outer membrane E1 glycoprotein, resulting in an amino acid change at position 226, which makes it easier for *A. albopictus* to disseminate the virus [24]. Because, *A. albopictus* is an endemic mosquito that is found practically everywhere, unlike *A. aegypti*, which is only prevalent in the tropics and subtropics [25]. In 2005–2006, one-third of La Reunion Island residents from France, and in 2013, the Caribbean population were suffered from CHIKV outbreaks, which led to the peril to public health in Western Europe and Central America [26,27,28]. These two outbreaks have caused hundreds of casualties and more than a million people have fallen ill [29]. Argentina, Mexico, Brazil, Bolivia, Netherlands, Colombia, Panama, Ecuador, Papua New Guinea, Federated States of Micronesia, Italy, Peru, Jamaica, Venezuela, Paraguay, Latin, and North America also reported CHIKV outbreaks in European and western hemisphere [30,31,32,33,34,35,36,37].

Climate change and shifts in vector and host populations may have increased CHIKV’s geographic expansion in addition to potential alterations enhancing viral fitness and transmissibility. Economic growth can’t shield nations from vector-borne illnesses; rather modern living may exacerbate an outbreak through travel, the aging of the population, and the accumulation of solid waste that can serve as a mosquito breeding ground. The United States armed force has acknowledged that CHIKV may be a biological weapon due to its biosafety level 3 (BSL3) pathogenicity. The National Institute of Allergy and Infectious Disease (NIAID) in the United States has designated it as a priority pathogen in category B, which ranks as biological agents’ second-highest category [38]. The WHO ranks CHIKV in the Research and Development Blueprint for preparedness for priority diseases/pathogens because further spread and epidemics are probable [39].

Epidemiological evidence indicating that a single CHIKV infection gives lifetime immunity against all the current lineages raises the possibility that only one vaccination may be enough to guarantee lifetime immunity [40]. Therefore, vaccination is still the most effective method for containing widespread CHIKV epidemics. The first CHIKV vaccine was said to be under development more than 50 years ago. As of now, preclinical testing has been conducted on at least 30 vaccine candidates and of which nine have advanced to clinical trials including BBV87 from Bharat Biotech International Ltd. (BBIL). Despite this, there are currently no licensed vaccines or antiviral medications available to treat CHIKV.

Ergo, this review focuses on the current status of biomedical research on CHIKV in the most populous country, India, and its epidemiology, clinical features, laboratory findings, management, and prevention adopting varied treatment strategies, with reference to the researchers and their affiliated institutions.

## 2. Organization of the CHIKV Genome

**Figure 1:**
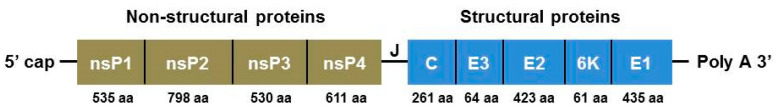
Schematic representation of CHIKV genome: four non-structural genes (nsp1–4), five structural genes including (Capsid, E3, E2, 6K, and E1) and junction region (J). The genome has a 5’ cap and 3’ poly A tail.

The linear, positive-sense, single-stranded genome of the RNA alphavirus CHIKV is approximately 11.8 kb in size [41]. It encompasses two open reading frames, one of which codes for four non-structural proteins (nsP1, nsP2, nsP3, and nsP4), and the other codes for five structural proteins, including the capsid (C), the peptide 6K/TF, and three envelope proteins (E1, E2, and E3) (Figure 1) [42,43]. The nsP123 precursor and nsP4 work together to synthesize full-length negative-strand RNA intermediate, and then nsP123 is sequentially processed into each of its constituent proteins to produce positive-strand genomic RNA (49S) transcription and sub-genomic RNA [44]. nsP1 possesses RNA capping characteristics and participates in the production of the viral RNA’s negative strand. Whereas the shut-off of host cell transcription is aided by the actions of nsP2 that possess RNA helicase, RNA triphosphatase, and proteinase activities. The viral RNA polymerase is nsP4, and nsP3 is a component of the replicase unit [45]. The capsid and envelope glycoproteins (E1 and E2) that make up the viral particle are contained in the structural polyprotein, which is translated from 26S sub-genomic RNA [44]. A lipid bilayer host-derived envelope securely encases the viral RNA, which is encapsidated in a nucleocapsid of approximately 40 nm in size and displays the glycoproteins E1 and E2 of the viral envelope [46]. The glycoproteins are divided into 80 trimeric spikes, with three E2/E1 heterodimers in each spike [47]. These spikes are necessary for the budding and growth of new virus particles, recognition of host receptors, and entries of cells through pH-dependent endocytosis (via E1) [48]. The class II fusion protein E1 facilitates membrane fusion that is brought on by low pH during viral infection. The type I transmembrane glycoprotein E2 interacts with the receptor during the alphavirus life cycle [47]. During the translation of the structural polyprotein (C–pE2–6K–E1), the capsid protein C is autocatalytically separated from it, and encapsidation of cytoplasmic viral genomic RNA occurs. The remaining envelope polyproteins are processed in the endoplasmic reticulum (ER) of the host cell and are processed by the host signalases at the N- and C-terminal ends of the 6K peptide to produce E3-E2 (also known as precursor E2 or PE2), 6K and E1 [48]. E3-E2 and E1 will eventually unite to form heterotrimers in the early Golgi compartment, following the proteolytic cleavage and it is exported to the plasma membrane. Subsequently, the E3-E2 goes through furin-dependent cleavage and brings out E3 from E2, which makes the trimeric spike fusogenic [49]. Alphavirus 6K has been linked to the processing of envelope protein, virion assembly, membrane permeabilization, and virus budding, whereas alphavirus E3 has been linked to the maturation of envelope glycoprotein [50]. The sequence-specific interaction of both 6K and PE2 or E1 leads to the formation of an efficient virus budding [51]. 6K facilitates lipids from the membrane to flip from one side of the bilayer to the other, during virus budding. Most of the time, 6K is not integrated into new virions; nonetheless, changes in 6K result in an impaired capacity of virions to operate in fusion and core deformation [52]. CHIKV can also be transmitted through cell-to-cell transmission, which enables virions to prevent attacks from the host immune system effectively [53].

## 3. CHIKV Research: Indian Scenario

### 3.1. Epidemiological Profile of CHIKV in India

The first CHIKV outbreak in India was recorded in the city of Kolkata in 1963, followed by Chennai, Puducherry, and Vellore in 1964 [54]. Retrospective serological tests, however, have revealed that chikungunya was present in India before 1963 [55]. After that, Visakhapatnam, Rajahmundry, and Kakinada in 1965, and the central part of India (Nagpur and Barsi) in 1973 were reported [56,57]. The virus reappeared in December 2005 after a 32-year gap, and it was actively spreading, infecting the Hyderabad district of Telangana and the Ananthapur district of Andhra Pradesh in South India, and ultimately affecting 1.4 million people in 13 states [58]. The worst-affected areas were Kerala state and Ahmedabad city in Gujarat [28]. By March 2006, 237 people had died and one-third of the Indian population was infected [29,59]. Until the 1970s, the source of CHICKV infection in India was due to the Asian genotype whereas the 2005–2006 outbreak was due to the ECSA genotype [60,61]. The illness spread to 15 states in 2009. By the years 2015, 2016, and 2019 it increased to 23, 28, and 30 states/Union territories respectively and the most laboratory-confirmed cases were reported in the years 2016, 2017, and 2019, in that order. The states of Maharashtra, Delhi, and Karnataka reported the most cases that were confirmed. A total of 81,914 clinically suspected CHIKV cases were reported in 2019, of which 12,205 (14.9%) were laboratory-confirmed cases in 21 Indian states and 3 Union territories. Karnataka reported the majority of CHIKV cases (3664), followed by Maharashtra (1646) and Telangana (1358) [62]. Till December 2019, Jammu and Kashmir, Mizoram, and Manipur reported 10 or fewer CHIKF cases [63,64]. Less spatial and temporal distribution of mosquitoes in the cold climate and lower urbanizations were major factors [65,66]. Based on the reports, there is a typical pattern of lower percentage positive values reported during the summer seasons, which rises when the rainy season begins, and stays high through the winter [67,68]. 2812 (15.2%) cases were laboratory confirmed as CHIKF until July 2020 out of a total of 18,533 clinically suspected CHIKV cases in 2020 [67]. CHIKF is a serious public health issue in our nation as it is still endemic in 22 Indian states and 4 union territories.

There have always been significant difficulties in the epidemiological surveillance of CHIKV infection, for instance, it can be challenging to distinguish CHIKF from other arbovirus-related ailments because of the similarities in their clinical symptoms or associated co-infections. Therefore, the severity of chikungunya is underestimated. Only precise serological and molecular assays measuring either the presence of anti-CHIKV antibodies in convalescent patients’ serum or the presence of CHIKV’s viral RNA can accurately diagnose CHIKF. To provide a more accurate assessment of incidence and prevalence, affected areas require more resources and surveillance. Nonetheless, there were only two virological testing laboratories in August 2006, the National Institute of Communicable Diseases (NICD) and the National Institute of Virology (NIV) for the entire country [69].

**Figure 2:**
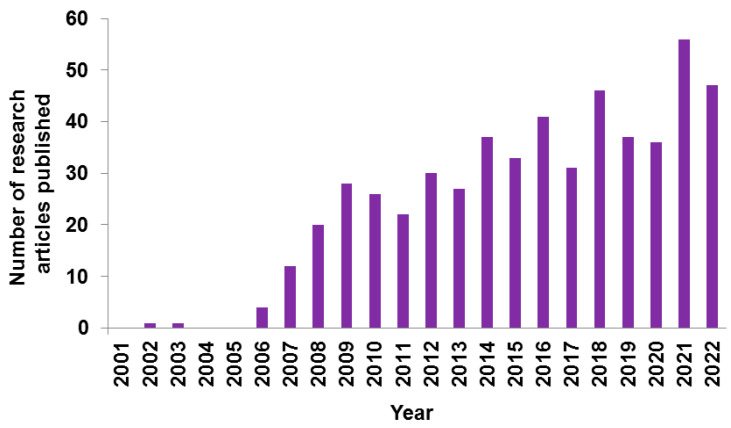
Number of year-wise publications on Chikungunya from India, in PubMed Database.

Over the past few decades, CHIKV outbreak studies have become popular in India’s research field to study how it spreads and affects the various populations living in different climatic, cultural, and environmental conditions [70]. A graphical representation of the year-wise publication on CHIKV research is presented in Figure 2. The major CHIKV research groups from India, based on their number of publications and the reputation of their affiliated institutions are summarized in Table 1.

### 3.2. Genomic and Proteomic Aspects of CHIKV

The genomic and proteomic data of CHIKV are vast. The comparison of various CHIKV strain genomes and the study of their genomic and proteomic relations give a reasoned knowledge of genetic diversity and phylogenetic relationships of CHIKV. Sreekumar et al., sequenced the 11.7 kb entire genome of the virus and genetic variants from six different viral isolates (2006 RGCB03, RGCB05; 2007 RGCB80, RGCB120; and 2008 RGCB355, RGCB356) from three consecutive Chikungunya epidemics in Kerala were examined [71]. Through full genome sequencing, Arankalle’s team investigated the relationship between the Indian isolates (IND-06) and Reunion Island isolates (RU) of CHIKV genomes, typical of various Indian states. The comparative genomic analysis of CHIKVs in India (1963–2006) with a reference to the 2005–2006 stormy epidemics has shown 99.9% nucleotide similarities. Three distinct substitutions were found in IND-06 isolates (2006): two for the Nsp1 region (T128K and T376M) and one for the capsid protein region (P23S) [72]. This team also verified that the CHIKV strain reported from India in 2000 shares 99% of its characteristics with a Ugandan strain from 1982. They concluded that the progenitor of the 2005–2007 CHIKV isolates originated from the neighborhood of Uganda [73].

Singh et al., reported that the ECSA genotype of CHIKV caused an epidemic between 2006–2010 and 2010–2014 in India [74,75]. Kumar and colleagues carried out a preliminary investigation to characterize genotyping of ECSA strain CHIKV involved in the outbreak of Kerala state and Puducherry. The Puducherry samples showed a mutation on K211E and Kerala samples showed a mutation on A226V in the CHIKV-E1 gene [76,77]. The ECSA genotype’s circulating CHIKV strains belonged to the Indian Ocean Lineage (IOL) category. But only a few different amino acids from the African strain S27 (AF369024) were found in the E1 polypeptide [78]. The crucial non-synonymous C/T mutation at position 10,670 in the E1 gene sequences from *A. albopictus* human isolates resulted in the alteration of the A226V amino acid, as mentioned *vide-supra* [79]. Santhosh from DRDE also disclosed E1: A226V shift in 2007 Indian CHIKV isolates [80]. Later, a team led by Dash from DRDE revealed new mutations namely E1:K211E and E2:V264A in CHIKV and it increased the replication and transmission effects of viruses in mosquitoes [81,82] and the same was corroborated in the clinical samples by Sunil’s group from ICGEB [83]. At the same time, the CHIKV-E2 K252H, another novel mutation, confirmed higher viral infectivity from a strain isolated from Kerala (2012–2013) [84]. Harsha et al., discovered yet another six non-synonymous mutations (D284E, V322A, K211E, M269V, V2201, and 1317V) in the CHIKV E1 gene between 2015 and 2016 [85]. By using double locus sequence typing, Muruganadam et al., identified the ECSA strain of the virus as being present in the Andaman and Nicobar Islands [86]. Whereas through E1 gene sequence analysis, Paramasivan et al., validated the involvement of Central/East African genotypes of CHIKV in the Lakshadweep Islands [87]. Dutta et al., also studied on ECSA strain and reported the variation of virus copy number from clinical symptoms among infected people [88]. They were also interested to look at toll-like receptor polymorphism associated with viral infection susceptibility and epitope prediction *in-silico* [89,90]. Chaaithanya’s team also carried out the gene cluster analysis of oligoadenylate synthetase and polymorphisms of the DC-SIGN (CD209) gene with patients’ symptoms due to CHIKV infections and also reported allele polymorphism of HLA class II in an outbreak from Andaman Island [91,92].

The amino acids from nsP2 (1–95) and nsP1 (170–288) are responsible for their straight interaction with ATPase enzymes demonstrated through mutational analysis [93]. The genetic analysis of nsP2 (378 bp), the E1 envelope protein (505 bp), and E2 (428 bp) by partial sequencing explained a mutation at L210Q (amino acids E2 200–220) in the coding region of E2.c [94]. The relationship between phylogenetic clade diversification and variations in in vitro infectivity among cosmopolitan CHIKV genotype strains was clarified by his team. They had novel mutations in the nsP1 (R171Q), nsP2 (I539S), nsP3 (N409T), and E2 (N72S) [84]. According to a study conducted by Jain and colleagues, the 2010 outbreak was the only one to have mutations in the nsP1 (G230R) and nsP3 (opal 524R) proteins, and these mutations were linked to the severity of the disease. Further, all of the sequenced 2016 samples included the nsP2 mutation H130Y [95].

Chaaithanya et al. investigated the role of chemokines and proinflammatory cytokines in CHIKV-induced chronic arthropathy and discovered that the main functions were played by the cytokines IFN-γ/β, IL-1, TNF-αl, MCP-1, IL-6, MIP-1β, and MIP-1α [96]. Few unusual reports of chronic arthropathy (CAR) and acute flaccid paralysis (AFP) following CHIKV infection were noticed in Andaman and Nicobar Islands, due to MIF gene polymorphism [97]. According to another study, conducted by Agarwal et al., mosquito saliva-induced cutaneous events promote the replication and progression of CHIKV, which is ensued by the induction of the anti-inflammatory genes IL-4, and IL-10 and the suppression of pro-inflammatory genes like IL-2, TLR-3, TNF-α, IFN-γ, and IFN-β is responsible for it [98]. According to Nair et al., with Ddx60, Usp18, Ifit1, Ifi44, Stat1, Rtp4, Mnda, Gbp3, Oas1g, Ly6a, Igtp, Gbp4, Gbp7, Oasl2, and Gbp10, the interferon-regulated gene (IRG) plays a significant role in CHIKV neurovirulence in the mice model [99,100].

Non-structural proteins nsP2 and nsP3 demonstrate RNA interference (RNAi) suppressor activity in the CHIKV proteins, according to research by Sunil’s team [101]. Through the use of next-generation sequencing techniques, this team identified 126 miRNAs from the *A. aegypti* cell line Aag2 that can control CHIKV replication, including miRNAs miR-100, miR-989, and miR-2b [102]. The same group also reported that the ubiquitin-related modifier is regulated by the microRNA miR-2b, which in turn regulates CHIKV replication and expansion in vitro [103,104]. Further, they investigated the transcriptome analysis and oxidative stress of vectors in response to DENV-2 and CHIKV mono- and co-infections [105,106]. In a coevolution study of CHIKV nonstructural proteins conducted by the same group, when each non-structural protein was taken into account separately, revealed 30 aa pairs coevolving in nsP1, 23 aa pairs coevolving in nsP2, 239 in nsP3, and 46 aa coevolving pairs in nsP4 [107]. Later, Parashar’s team studied altered microRNA expression in the CHIKV-infected fibroblast cells from the mammalian system and purported the possible use of let-7e, miR15, miR-16, miR-17, miR-23a, miR-99, and miR-125 as a biomarker in the viral infection [108].

A bioinformatics team led by Cherian from NIV examined the evolutionary rates and timeline comparability of CHIKV as inferred from the complete genome/E1 gene, paying particular attention to the early outbreaks (2005–2007) in the area bordering the Indian Ocean. The evolutionary timescale of CHIKV was calculated to be in the last 30 decades under a constant population relaxed clock model in bioinformatics [73]. This team also characterized the E1 and E2 genes of CHIKV collected from various parts of India during the 2015–2017 outbreaks [109]. The “Genome to Hits In Silico” strategy was proposed by Jayram’s team, who collaborated with a group of biologists from IIT Delhi, to illustrate novel CHIKV genome-based pathways. This strategy integrates several steps, including gene prediction, determination of the protein’s tertiary structure, identification of the active site, generation of hit molecules, docking of hits, and scoring of hits to arrive at lead compounds, adopting *in-silico* method [110,111,112].

Sreekumar and colleagues were involved in proteomic analysis and viral replication studies by CHIKV infection, adopting in vivo and in vitro systems [94,113]. Upon CHIKV infection in mammalian cells, their proteomic investigation of the nuclear and multifunctional chaperone nucleophosmin (NPM1)/B23 revealed non-permanent regulation and cytoplasmic aggregation, which limits viral multiplication in the host cells [114,115]. Proteomic analysis of CHKV-infected newborn mouse tissues was performed by Parida’s group and they discovered radical physiological reprogramming through differentially produced proteins from various classes, including those involved in stress, inflammation, apoptosis, urea cycle, cytoskeletal, lipid, and energy metabolism pathways of disease pathogenesis [116,117]. Through the proteome profiling of serum samples from virus-infected individuals, Pandey and his team provided information based on host reaction. During viral infection, the apolipoproteins, clusterin, and S100A family of proteins are active host response factors [118].

### 3.3. Diagnostics, Treatment, and Vaccines for CHIKV

#### 3.3.1. Diagnostics

The lack of well-developed diagnostic techniques, licensed drugs, vaccines, and antiviral therapeutics led to the primary outbreaks of CHIKV in India. Usha’s group studied similarities between dengue and CHIKF and their co-infection [119] and they concluded that Rheumatologists should always consider Chikungunya infection as one of the causes of arthralgia, after finding seroprevalence of anti-chikungunya IgG antibodies among Rheumatoid Arthritis patients [120]. A research group led by Vijayachari revealed that CHIKV infection can change joint morphology in acute and chronic arthritis and is explained by the imaging technique [121]. Interestingly, four cases of CHIKV infection showed flaccid limb weakness with high body temperature, myalgia, bony erosion, arthralgia, and arthritis, and in a few cases maculopapular rashes in Andaman and Nicobar Islands. They concluded the infection of the virus by serological method (IgM ELISA method) [122,123].

Based on an examination of a variety of ocular symptoms in a cohort of CHIKV-infected individuals, conducted by Prajna, an ophthalmologist, the primary ocular symptom of the CHIKV infection included granulomatous and non-granulomatous anterior uveitis, retrobulbar neuritis, optic neuritis, and dendritic lesions [124]. Mahendradas, another ophthalmologist also reported various ocular manifestations associated with CHIKV infection [125,126,127,128]. The anterior uveitis, retinitis, and optic neuritis were the commonest manifestations, and a case of bilateral Fuchs’ heterochromic iridocyclitis was also reported [129,130]. Yet another ophthalmologist, Mittal also explained optic neuritis and uveitis associated with CHIKV infection in South India [131,132]. Further, Rose et al., noticed retrobulbar neuritis, bilateral papillitis, perineuritis, and neuro retinitis in CHIKV-infected individuals [133]. When Riyaz, a dermatologist reported cutaneous manifestations including purpuric macules with vesiculobullous lesions due to CHIKV infection [134,135,136], Bandyopadhyay observed hyperpigmentation, aphthous-like ulcers, excoriated papules, erythema, xerosis, vesiculobullous and lichenoid eruptions in the skin [137,138,139]. Kumar et al. concluded that anyone with painful oro-genital and intertriginous aphthous-like lesions linked to febrile polyarthralgia with a rash should be suspected of having the CHIKV virus [103]. In another study of febrile chikungunya patients, Murali-Krishna and colleagues described the antibody response patterns that had developed during the febrile phase and these differences could be used as biomarkers to predict future protection from or progression to chronic arthritis [140]. In yet another study of the clinical characteristics of CHIKV-infected patients at a tertiary care facility in Maharashtra, Dube identified some uncommon problems such as lymphadenopathy, mouth ulcers, and encephalitis [141]. Further, Lodha and colleagues discussed CHIKV infection in children and observed the same clinical features as elders [142,143]. However, Patel et al. were interested in studying the acute phase of CHIKV infection in infants and explained how the viral load causes clinical features in kids [144,145]. The assessment of CHIKV infection in Indian children conducted between 2009 and 2010 revealed that the southern region of India had a higher percentage of CHIKV infection than the northern region.

When Patil et al. assessed the level of CHIKV exposure by estimating the age-stratified prevalence of IgG antibodies [146,147], Ravi from NIMHANS studied the association of plasma viral loads and the presence of chikungunya IgM antibodies during acute CHIKV infection with cytokine/chemokine levels [148]. For CHIKV diagnosis, IgM ELISA, TaqMan Real-Time PCR, RT-LAMP assay, and reverse transcription PCR were employed [149]. Lakshmi et al. also discussed the clinical signs and symptoms of CHIKV infection as well as various molecular diagnostic methods, such as the RT-LAMP assay [150]. A single-step multiplex real-time RT-PCR technique for simultaneously detecting and quantifying the RNA of both the DENV and the CHIKV was developed by Cecilia’s dengue/chikungunya group from NIV and it worked well for CHIKV and DENV differential diagnosis [151]. IgM ELISA, nested RT-PCR, and real-time quantitative PCR (qPCR) were evaluated by Parashar’s lab as well, for their ability to diagnose CHIKV, with both assays being ideal for definitive diagnosis [152]. Besides, Tomar and colleagues from IITR recently developed tamarind chitinase (chi)-like lectin glycan-based virus capture assay as a point-of-care routine diagnostic and titration assay for CHIKV as well as for other re-emerging alphaviral infections [153]. Moreover, they recently reported that exploiting NLuc CHIK-VLPs as a detector probe offers the potential for large-scale screening of samples [154]. Further, Sunil’s group evaluated the immunochromatographic rapid diagnosis kit for the detection of CHIKV antigen in the country and performed serological, virological, and clinical analyses of people infected with the virus [64,155,156,157]. Ray’s group succeeded in preparing CHIKV-E2 recombinant protein in *E.coli* via cloning [158]. Adopting ELISA or Western blot, this isolated protein can accurately be utilized to identify CHIKV-specific antibodies in patients’ sera who had PCR or IgM-positive results [159,160,161]. When the truncated E2 protein was employed for antibody detection, they recently discovered a considerable overlap between CHIKV-infected participants and those with other febrile diseases [161]. Interestingly, when binding antibody titers were determined using fixed CHIKV particles as the test antigen, this overlap was more pronounced. Whereas, the *in-vitro* identification of the key molecules and pathways involved in CHIKV infection is made possible by the combined mRNA and miRNA signature by Saxena et al. [162].

A research team led by Parida from DRDE developed and analyzed the antigen capture ELISA for CHIKV early clinical diagnosis. Further, real-time RT-PCR assays for detection and quantification were also tested [163,164]. They also developed a quantitative competitive reverse transcription polymerase chain reaction (QC-RT–PCR) for the detection and quantitation of CHIKV [165]. Furthermore, the diagnostic ability of CHIKV viral antigens purified by recombinant envelope proteins and native cell culture was also evaluated [166]. In addition, they generated high-affinity E2-specific monoclonal antibodies against the recombinant protein E2 (rE2) of the CHIKV, and it was reported that these antibodies might be useful for early clinical diagnosis and cohort studies of the virus [167]. The recombinant GST-capsid protein was developed by prokaryotic expression, and ELISA and western blotting confirmed the immunoreactivity of the recombinant antigen. The clinical diagnostic examination of human patients demonstrated 100% sensitivity and 95% specificity in the identification of anti-CHIK IgM antibodies in the serum acute phase samples [168]. This team proved that the RT-LAMP test is a valuable technique for quick, real-time detection and CHIKV quantification in acute-phase serum samples [169]. Another team under the direction of Santhosh examined a one-step real-time RT-PCR assay based on SYBR Green I for the detection and quantification of CHIKV [170]. Besides, the nsP2 protease-based cell-free high-throughput screening assay was developed by Dash’s group to test inhibitors against emerging CHIKV [171].

#### 3.3.2. Treatment

Chloroquine is a popular anti-malarial drug with the potential for both prophylaxis and treatment of CHIKV [172]. Parida’s group looked at the in vitro prophylactic and curative effects of chloroquine on Vero cell CHIKV replication. Chloroquine’s suppressive effects include reduced endosomal-mediated virus entry during the early stages of virus replication, mostly by preventing endocytosis and/or endosomal acidification [173]. Based on another study conducted by the same team, the four artificial microRNAs effectively inhibit Vero cell CHIKV replication [174]. Further, they proposed that the inhibition of CHIKV replication, and apoptosis brought on by viruses in cultured mammalian cells may be accomplished by inhibiting the activity of the cellular inosine monophosphate dehydrogenase (IMPDH) enzyme [175]. Moreover, their study of the effectiveness of combining RNAi-based therapy with traditional antivirals such as ribavirin, chloroquine, and mycophenolic acid provided insight into the decision to use RNAi-based combination therapy [174]. Chattopadhyay and colleagues studied the effect of Ibuprofen conjugated with sulfonamide and thiosemicarbazide and found that the conjugation imparted anti-CHIKV properties while maintaining the anti-inflammatory attributes of Ibuprofen [176].

According to Parashar et al., the administration of NS1 siRNAs and E2 can suppress CHIKV replication *in-vitro* and it can protect mice infected with the virus [177]. To create novel antiviral strategies, they also attempted to identify and describe the relationship between the host and the virus. Through the use of the viral overlay protein binding assay (VOPBA) and matrix-assisted laser desorption/ionization-time of flight analysis (MALDI TOF/TOF), HSP70 and actin were discovered to be virus-binding proteins in HEK-293T and Vero-E6 cells [178]. Chattopadhyay’s team compared the infection pattern of the Indian CHIKV outbreak (2006), the DRDE-06 strain, and the S-27 prototype strain in mammalian cells [179]. When these two viral strains are infected, the heat shock protein 90 (HSP90), which stabilizes nsP2, positively influences CHIKV proliferation [180]. 17-AAG, a putative HSP90 inhibitor, can significantly control viral infection, apoptosis, and the generation of pro-inflammatory cytokines and chemokines in the host macrophages [181].

Based on conformer and pharmacophore, Tomar and his team at the IITR discovered the peptidomimetic inhibitors of CHIKV-nsP2 protease. The molecular docking of the nsP3/4 junction peptide at the CHIKV nsP2 protease active site gave structural insight into the nsP3/4 peptide’s likely mode of binding and identified key molecular interactions for the substrate binding mode [182,183,184]. Besides, they showed that the peptidomimetic inhibitors Pep-I and Pep-II target virus-specific cysteine protease for CHIKV suppression [185]. Through a further study of CHIKV-nsP2 cysteine protease crystal structure, they have shown its active site with a putative flexible loop blocking (nsP2pro), and this might be beneficial for structure-based drug designing [186,187]. Tomar and her team also investigated the trans-proteolytic activity of the CHIKV capsid protease (CVCP) and discovered that it is crucial to create inhibitors against CHIKV enzymes to obstruct crucial steps in viral multiplication [188,189]. Furthermore, they reported that Piperazine and picolinic acid (PCA) suppresses viral multiplication in Vero cells by attaching to the hydrophobic pocket of the CHIKV capsid protein [190,191]. Moreover, the antiviral activity of NAG-specific chi-like lectin revealed the glycan-dependent chikungunya viral infection [192]. In terms of potential entry inhibitors for CHIKV, phenothiazine, bafilomycin, and 4-hydroxy-1-methyl-3-(3-morpholinopropanoyl)quinoline-2(1H)-one may be regarded as the lead drugs [193] and the same was revealed by structural modeling, molecular dynamic and in silico docking studies [194]. On top of that, Moralbanone, Kaempferol, and Rutin have been reported by Ray’s group to have better binding potential with CHIKV nsP2 and E1 proteins through in silico studies [195].

Dilip et al. compared different medical ideologies (such as ayurveda, allopathy, homeopathy, etc.) and investigated how conventional healthcare professionals treat CHIKV infections [196]. In southern India, people are using Dhanvantaram Gutika, Vilwadi Gulika, Sudarsanam Gulika, Vettumaran Gulika, Amruthotharam Kashayam, Amritarishta, Amruthadiguggulu, Rasnaeranda Kwatha Churna, Vanathulasi Patra and *Chromolaena odorata* as ayurvedic drugs to treat CHIKV infections. Homeopathic medicines like Eupatorium Perfoliatum Mother Tincture, Rhus tox, Influenzinum, Pyroginum, Arnica, Cedron, Belladonna, Ledumpal, Bryonia, and Nuxvomica are also used against CHKV [196]. The anti-CHIKV activities of *Vitex negundo*, *Hyptis suaveolens*, and *Decalepis hamiltonii* are reported by Swaminathan [197]. Brahmanandha Bairavam Mathirai and Vishnu Chakram, two polyherbal siddha formulations were tested and found to be effective against CHIKV infection by Sunil from ICGEB [198]. The ethanolic extract of Nilavembu kudineer, aqueous and ethanolic extracts of the *Vathasura kudineer*, and the traditional Indian remedy Amukkara Choornam (*Withania somnifera*-based) exhibit antiviral properties against CHIKV [199,200,201]. Besides, Patel’s lab from IIT Delhi also evaluated the anti-CHIKV activity of medicinal herbs. They identified that *Cedrus deodara*, *Picrorhiza kurroa*, *Terminalia chebula*, *Ocimumtenui-florum*, and *Commiphora wightii* plant extracts exhibited inhibition of CHIKV attachment via helicase and protease inhibition [202]. This team used an FDA-approved chemical library and an *in-silico* drug screening method to find inhibitors of the CHIKV-nsP2 protease, a crucial non-structural protein needed for the reproduction of the CHIKV virus. The antibiotic novobiocin and the antihypertensive medication telmisartan were among the top hits on the screen and were found to be significant inhibitors of viral replication [203,204].

#### 3.3.3. Vaccine

Due to the pressing need for vaccinations to protect against various viral illnesses, the Indian government launched the National Immunization Program, which resulted in the establishment of numerous authorized vaccine manufacturing facilities around the nation. Vaccine institutes were established at the beginning of the 20th century, and they gradually evolved in the direction of privatization, like BBIL. Besides BBIL, a few Indian agencies are fervently working on the CHIKV vaccine, including the Defense Research Development Establishment, the National Institute of Virology, and the Serum Institute of India.

An ILS researcher Chattopadhyay intended to comprehend anti-CHIKV CD8+ T cell response and epitope advancement-based immunotherapy for CHIKV infection, in silico [205]. Polyclonal antibodies against synthetic peptides of CHIKV non-structural proteins were produced and characterized [206] and it was discovered that the monoclonal antibodies are very sensitive and specific for CHIKV-nsP2 and stop viral multiplication [207]. Arankalle’s group aimed to evaluate envelope-specific immune responses in individuals with CHIKV infection in 2006. The findings demonstrated that lower levels of IFN-γ may be associated with the severity of the disease in these individuals, and T cell epitopes recognized on the envelope region of CHIKV by all patients [208,209]. This team intended to create potential vaccines using either recombinant CHIKV-E2 protein or chemically inactivated whole CHIKV. They showed that the rE2p with alum adjuvant and the BPL/formalin-inactivated CHIKV are promising potential vaccines to prevent the infection [210]. Recently Rahatgi’s group from IITR provided a discernment of CHIKV E2-FL protein immunogenic epitopes, which can be used for the development of novel multiepitope-based anti-CHIKV vaccine strategies [211]. Kumar’s team conducted an in silico study for the development of a CHIKV epitope-based vaccine as well as diagnostic development [212]. Sreekumar and colleagues reported through another recent study the probable application of an attenuated CHIKV strain RGCB355/KL08-p75 as a probable candidate for vaccine development [213]. Using bioinformatics tools, Slathia’s team predicted the DNA vaccine design for CHIKV, based on the conserved T and B cell epitopes derived from structural protein [214]. The ECSA genotype of CHIKV provided the immunogenic potential of Vero-adapted formalin-inactivated vaccines, which were shown to have immunogenic potential to reduce the rate of virus infection by enhancing both humoral and cell-mediated immune response [215]. Further, using a novel yeast expression system (*Pichia pastoris*), chikungunya virus-like particles (CHIK-VLPs) were created by Parida’s team and tested as a potential vaccination candidate in Balb/c mice. Through the stimulation of TNF-α, IL-10, and significant quantities of IL-4, IL-2, and IFN-γ, a higher level of cellular immune response was seen, indicating a regulated response [216]. Furthermore, they investigated the immunogenic capacity of different adjuvants and recombinant CHIK-E1 and CHIK-E2 proteins produced in bacteria. Studies on neutralization conducted both in vitro and in vivo have verified the evaluation of the protective efficacy of the vaccine formulations [217].

Sumathy from BBIL looked at evolutionary dynamics and serotype analyses [218,219]. Based on the ECSA strain, they developed a pure, inactivated CHIKV immunogenic formulation (BBV87) that may be used as a vaccine to prevent viral infection [220,221]. Formalin, which is frequently used to produce inactivated viral vaccines and has also been tested in preclinical studies with CHIKV, is employed in the BBV87 vaccine as the technique of viral inactivation. The BBV87 vaccine was discovered to have the capacity to induce a sufficient immune response against CHIKV in an Indian phase I clinical research. BBIL submitted the study in 2017 to the Clinical Trial Registry-India and it has recently begun Phase II and III clinical trials in Costa Rica, according to a recent announcement from the International Vaccine Institute (IVI), a partner of BBIL in this endeavor. It was supported by the India Centric Epidemic Preparedness (Ind-CEPI) mission of the Department of Biotechnology, Government of India, and sponsored by the Coalition for Epidemic Preparedness Innovations (CEPI). Phase II/III clinical trials beginning in Costa Rica, and by others are key steps towards reaching the objective of quick vaccine development of Ind-CEPI.

### 3.4. Biotechnological Aspects of CHIKV

The function of male *A. aegypti* mosquitoes in the maintenance and transmission of CHIKV to female mosquitoes was demonstrated by Mishra’s team at NIV. They verified that CHIKV can spread to female mosquitoes during mating by infected male mosquitoes. Infant Swiss albino mice became sick after being exposed to the body fluids of venomous female mosquitoes, demonstrating the infectiousness of the venomous virus [222]. Another study from the same institution, by Mourya’s team, investigated how temperature stress affected juvenile stages and *A. aegypti* mosquitoes’ sensitivity to CHIKV. They established that mosquitoes perished at temperatures above 44.5 °C, which had an impact on the propagation of CHIKV [223]. By employing *A. aegypti* rosy eye mutants, they were able to determine the genetic basis of the virus’ vector competence for CHIKV [224]. The significance of the gregarine parasite *Ascogregarina culicis* in the maintenance of CHIKV in vector mosquitoes was also examined by this group. They observed that the oocyst of the parasite in the *A. aegypti* mosquitoes directly transmits the CHIKV virus vertically. It has been hypothesized that *A. culicis* may play a significant part in CHIKV maintenance throughout the inter-epidemic period [225]. Yadav et al. observed the potential CHIKV-specific receptor proteins on the midgut brush boundary membrane of *A. aegypti*, and carried out experimental CHIKV transmission via *Anopheles stephensi* mosquitoes [226,227]. They verified that *A. aegypti* infected with CHIKV showed post-inoculation alterations in the enzyme activity of acetylcholinesterase, glutathione-S-transferase, glucose-6-phosphate dehydrogenase, and non-specific esterases [228]. The team also attempted to study an in-depth perspective of the potential of Zika virus infectivity to already infected *A. aegypti* with DENV and CHIKV and the results proved that it multiplied effectively in the mosquitoes [229]. Ravi from NIMHANS revealed the ultrastructural morphological changes of host cells by the infection of CHIKV [230]. The pathogenesis is influenced by the CHIKV E1 protein’s molecular mimicry of host proteins [231]. Desai led a different team from the same institute that looked into the mechanisms underlying the three kinds of *Aedes mosquitoes’* ability to serve as CHIKV vectors [232]. To enter the mosquito cell line, the CHIKV interacts with the protein heat shock cognate 70 (HSC 70), and C6/36 cells treated with quercetin and YM-01 showed dose-dependent inhibitory potential [233]. Sunil examined the effects of CHIKV replication on *A. aegypti’s* life table features and the pathways involved in egg-laying [234].

## 4. Research Priorities and Future Directions

Despite the earnest attempts and technological advancements, multiple concerns about CHIKV infection remain yet to be addressed. Point-of-care multiplex diagnostics to diagnose arboviruses are needed urgently, given the co-circulation of these viruses, including more specific serological assays for seroprevalence studies. More research and mitigation measures are required for the socioeconomic and environmental factors, that contribute to vector proliferation, particularly in low-income cities. Reasons behind the dramatic outbreaks in naive areas need to be unveiled. One of the economical and long-lasting approaches for reducing the disease is to combine therapies that are effective against several arboviral diseases. Lack of funding is a main crisis for the development of a successful CHIKV vaccine, although active immunization is the most economical preventative strategy. Therefore, there is an urgent need for global partnerships with industry, researchers, clinicians, public health officials, and other stakeholders in global health. These partnerships must include national and international, public, private, and government funding agencies to resolve this crisis. The quick deployment of SARS-CoV-2 vaccines in 2020 showed that the scientific community and financing organizations can overcome the customarily slow pace of vaccine development. Nonetheless, numerous potential vaccination candidates are currently undergoing clinical testing and will hopefully start the marketing authorization procedure soon. Yet, it is challenging to conduct vaccine efficacy studies due to the spontaneous emergence of CHIKV infections during outbreaks. An establishment of serological correlates of protection would be an appropriate strategy to make the marketing authorization simpler. The efficacy of the candidate vaccines is still unknown in the pediatric population because they are currently developed primarily for the adult population. Therefore, this part also needs to be considered.

## 5. Conclusions

In India, CHIKV-related research has boomed during the last decades. According to our current data, the CHIKV-related research has concentrated on its varied aspects such as epidemiology, vector entomology, virology, clinical microbiology, cell biology, immunology, molecular biology, vaccinology, genetics, geo-medical and disease environmental studies, and disease burden estimation. Remarkably, hundreds of articles of high quality have been published by Indian researchers in peer-reviewed journals such as The Lancet, JAMA, Clinical Infectious Diseases, Journal of Medical Virology, Travel Medicine and Infectious Disease, Journal of Infection and Public Health, Virus Research and Scientific Reports. Following the resurgence of viral infectious diseases like Zika, Nipah, and the COVID-19 pandemic, there have been significant advances in surveillance, disease diagnostics like the CRISPR system, convalescent plasma therapy, and vaccine development like the mRNA vaccine. Nonetheless, as a priority pathogen of great concern due to its propensity to produce incapacitating illness, CHIKV is still regarded as a neglected tropical illness. The lack of funding, the unpredictable nature of the disease’s epidemiology, and the erratic interest in this pathogen, which has risen and fallen with the frequency of CHIKV outbreaks, have all hindered the development of CHIKV research progress. The loss of lives during the latest COVID-19 epidemic and the 1918 Spanish flu pandemic emphasizes the usefulness of vaccines in containing epidemics. Albeit a wide range of potential preclinical and clinical vaccine candidates have been developed in recent years, and it can be considered a milestone in the fight against this debilitating disease, there are presently no approved medicines or vaccines to prevent CHIKV infections. Ergo, this review briefs the CHIKV research in India and put forward the priorities and suggestions to favor a positive outcome. Hitherto, it is alluring to conjecture that in par with the world’s hastening CHIKV research, and heightened necessity for research and development of efficient medication and/or vaccine for this neglected tropical illness, Indian scientists will be able to contribute adequately and the cure will be available sooner than we anticipate.

## Figures and Tables

**Table 1:** Major Indian Biomedical Research Groups Working with the CHIKV.

No	Institute	Principal Investigator	Department/Area of Interest	Funding	Location
1.	AIIMS	Rakesh Lodha	Department of Pediatrics		New Delhi
2.	BBIL	K. Sumathy		BBIL	Hyderabad
3.	CRME	Brij Kishore Tyagi	Vector-borne Diseases	DBT, CSIR	Madurai
4.	CSTM	Anusri Tripathi	Molecular Biology & Molecular Genetics		Kolkata
5.	DRDE	Manmohan Parida	Division of Virology	DRDO	Gwalior
6.	DRDE	Paban Kumar Dash	Division of Virology	DRDO	Gwalior
7.	DRDE	P.V. Lakshmana Rao	Division of Virology	DRDO	Gwalior
8.	DRDE	S.R. Santhosh	Division of Virology	DRDO	Gwalior
9.	ICGEB	Sujatha Sunil	Vector Borne Diseases	CSIR, DBT, DST, ICGEB, Ministry of Ayush	New Delhi
10.	ICMR	Shyamalendu Chatterjee	Epidemiology	ICMR	Kolkata
11.	ICMR- RMRC	Paluru Vijayachari	Molecular Epidemiology and Clinical Microbiology	DBT, ICMR-RMRC	Port Blair
12.	ICMR-VCRC	Muniaraj Mayilsamy	Parasitology	ICMR	Madurai
13.	ICMR-VCRC	Narendran Pradeep Kumar	Vector Biology & Control	ICMR	Kottayam
14.	IIM	Dileep Mavalankar	Center for Management of Health Services Public Systems Group		Ahmedabad
15.	IITD	Ashok Kumar Patel	Virology	DST, DBT	New Delhi
16.	IITR	Shailly Tomar	Antiviral Research, Molecular and Structural Virology	DBT, DST, DRDO, ICMR	Roorkee
17.	ILS	Soma Chattopadhyay	Infectious Disease Biology	UGC, DBT, DST, ICMR	Bhubaneswar
18.	JH	Pratima Ray	Department of Biotechnology	DBT	New Delhi
19.	JMI	Shama Parveen	Evolution of Dengue and Chikungunya Viruses	UGC, CCRUM, DST, CSIR, ICMR	New Delhi
20.	Narayana Nethralaya Post Graduate Institute of Ophthalmology	Padmamalini Mahendradas	Ophthalmology		Bangalore
21.	NIE	Vidya Ramachandran	Impacts of CHIKV Outbreaks in Chennai	ICMR-NIE	Chennai
22.	NIE	Manoj V. Murhekar		ICMR	Chennai
23.	NIMHANS	Vasanthapuram Ravi	Department of Neurovirology		Bangalore
24.	NIRRH	Itta Krishna Chaaithanya	Vector Biology and Clinical Virology	ICMR, DBT	Mumbai
25.	NIV	Vidya A. Arankalle	Virology and Vaccinology	ICMR, NIV	Pune
26.	NIV	Kalichamy Alagarasu	Dengue/Chikungunya Group	SERB	Pune
27.	NIV	Akhilesh Chandra Mishra	Epidemiology and Control Planning of Vector-borne Diseases	ICMR	Pune
28.	NIV	Devendra Tarachand Mourya	Molecular Virology and Epidemiology	NIV	Pune
29.	NIV	Sarah S. Cherian	Bioinformatics and Computational Biology	NIV	Pune
30.	NIV	Deepti Parashar	Molecular Virology	ICMR	Pune
31.	RGCB	Easwaran Sreekumar	Molecular Virology Laboratory	DBT	Thiruvananthapuram
32.	RMRC	Prafulla Dutta	Arbovirology Group	ICMR	Dibrugarh

AIIMS—All India Institute of Medical Sciences; BBIL—Bharat Biotech International Ltd.; CRME—Centre for Research in Medical Entomology; CSTM—Calcutta School of Tropical Medicine; DRDE—Defense Research and Development Establishment; ICGEB—International Centre for Genetic Engineering and Biotechnology; ICMR—Indian Council of Medical Research; RMRC: Regional Medical Research Centre; VCRC—Vector Control Research Centre; IIM: Indian Institute of Management; IITD—Indian Institute of Technology Delhi; IITR: Indian Institute of Technology Roorkee; ILS—Institute of Life Sciences; JH—Jamia Hamdard; JMI—Jamia Millia Islamia; NIE—National Institute of Epidemiology; NIMHANS: National Institute of Mental Health and Neurosciences; NIRRH: National Institute for Research in Reproductive Health; NIV: National Institute of Virology; RGCB—Rajiv Gandhi Centre for Biotechnology; UGC—University Grants Commission; CSIR—Council of Scientific and Industrial Research; SERB: Science and Engineering Research Board; DRDO—Defense Research and Development Organization; DST: Department of Science and Technology; CCRUM—Central Council for Research in Unani Medicine.

## Data Availability

Not applicable.
